# CLIPB10 is a Terminal Protease in the Regulatory Network That Controls Melanization in the African Malaria Mosquito *Anopheles gambiae*

**DOI:** 10.3389/fcimb.2020.585986

**Published:** 2021-01-15

**Authors:** Xin Zhang, Miao Li, Layla El Moussawi, Sally Saab, Shasha Zhang, Mike A. Osta, Kristin Michel

**Affiliations:** ^1^Division of Biology, Kansas State University, Manhattan, KS, United States; ^2^Department of Biology, American University of Beirut, Beirut, Lebanon; ^3^Department of Entomology, China Agricultural University, Beijing, China

**Keywords:** innate immunity, melanization, serine protease, serpin (serine proteinase inhibitor), phenoloxidase

## Abstract

Humoral immune responses in animals are often tightly controlled by regulated proteolysis. This proteolysis is exerted by extracellular protease cascades, whose activation culminates in the proteolytic cleavage of key immune proteins and enzymes. A model for such immune system regulation is the melanization reaction in insects, where the activation of prophenoxidase (proPO) leads to the rapid formation of eumelanin on the surface of foreign entities such as parasites, bacteria and fungi. ProPO activation is tightly regulated by a network of so-called clip domain serine proteases, their proteolytically inactive homologs, and their serpin inhibitors. In *Anopheles gambiae*, the major malaria vector in sub-Saharan Africa, manipulation of this protease network affects resistance to a wide range of microorganisms, as well as host survival. However, thus far, our understanding of the molecular make-up and regulation of the protease network in mosquitoes is limited. Here, we report the function of the clip domain serine protease CLIPB10 in this network, using a combination of genetic and biochemical assays. CLIPB10 knockdown partially reversed melanotic tumor formation induced by Serpin 2 silencing in the absence of infection. CLIPB10 was also partially required for the melanization of ookinete stages of the rodent malaria parasite *Plasmodium berghei* in a refractory mosquito genetic background. Recombinant serpin 2 protein, a key inhibitor of the proPO activation cascade in *An. gambiae*, formed a SDS-stable protein complex with activated recombinant CLIPB10, and efficiently inhibited CLIPB10 activity *in vitro* at a stoichiometry of 1.89:1. Recombinant activated CLIPB10 increased PO activity in *Manduca sexta* hemolymph *ex vivo*, and directly activated purified *M. sexta* proPO *in vitro*. Taken together, these data identify CLIPB10 as the second protease with prophenoloxidase-activating function in *An. gambiae*, in addition to the previously described CLIPB9, suggesting functional redundancy in the protease network that controls melanization. In addition, our data suggest that tissue melanization and humoral melanization of parasites are at least partially mediated by the same proteases.

## Introduction

Melanization, the biochemical formation and deposition of melanin fulfills diverse biological functions in living organisms (Cerenius and Soderhall, 2004; Vavricka et al., 2010; Sugumaran and Barek, 2016; Pavan et al., 2020). In arthropods, including mosquitoes the production of eumelanin is a broad-spectrum immune response against viruses (Rodriguez-Andres et al., 2012), bacteria (Hillyer et al., 2003; Yassine et al., 2014), fungi (Yassine et al., 2012a), chromista (Brey et al., 1988), and other eukaryotic parasites (Christensen, 1981; Michel et al., 2005; Habtewold et al., 2008). In mosquitoes, melanization initially received much attention as a selectable phenotype that confers refractoriness to parasites of public health importance including, malaria parasites and filarial worms (Collins et al., 1986; Chen and Laurence, 1987; Hurd et al., 2005). Subsequent genetic studies identified single genes whose knockdown (kd) triggers a potent melanotic response against *Plasmodium* ookinetes (Osta et al., 2004; Michel et al., 2005; Frolet et al., 2006; Nakhleh et al., 2017a), drawing considerable attention to the potential application of this response in controlling vector competence. Indeed the non-vector mosquito, *Anopheles quadriannulatus*, was shown to trigger a potent immune response to *Plasmodium* ookinetes characterized by a significant melanization of *P. berghei* ookinetes and occasionally of *P. falciparum* (Habtewold et al., 2008).

The infection-induced melanization in *An. gambiae* is tightly regulated by the complement-like pathway, specifically the thioester-containing protein 1 (TEP1), which upon activation is deposited on the surface of entities that are recognized as damaged or foreign (Blandin et al., 2004; Yassine et al., 2012a; Povelones et al., 2013). TEP1 binding to microbial surfaces triggers, in a yet unknown mechanism, the activation of a complex downstream network of clip domain serine proteases (CLIPs) constituted of both catalytic proteases (cSPs) and their non-catalytic homologs (cSPHs). A terminal protease in the cascade ultimately cleaves and activates the zymogen prophenoloxidase (proPO) into active phenoloxidase (PO), the rate-limiting enzyme in melanogenesis (Nakhleh et al., 2017b; Rhodes and Michel, 2017; Bartholomay and Michel, 2018). Melanin formation on surfaces of microbes is thought to hinder their intake of nutrients, while toxic intermediates, such as reactive oxygen and reactive nitrogen species, may also cause cellular damage (Nappi et al., 2009).

There are 110 cSPs and cSPHs currently annotated in the *An. gambiae* genome, which are divided into five sub-groups called CLIPA-E, based on phylogeny, clip-domain structure, and domain arrangement (Christophides et al., 2002; An et al., 2011; Cao et al., 2017). Groups B-D largely contain cSPs, with one or more clip domains at the amino terminus and a chymotrypsin-like protease domain at the carboxyl terminus. In contrast, all members of the CLIPA family are cSPHs and contain one to five clip domains at the N-terminus and a protease homolog domain at the carboxyl terminus, while CLIPEs include several members containing one catalytic domain plus one or more protease homolog domains in the same molecule. CLIPBs are core components of CLIP cascades that are secreted into the hemolymph as zymogens and are activated sequentially by specific cleavage at the linker region between the clip and protease domains by an upstream protease (An et al., 2011; Zhang et al., 2016). Studies in other model insects have shown that CLIPCs act upstream of CLIPBs in these cascades and that the terminal conversion of proPO to active PO is mediated by prophenoloxidase activating proteins (PAPs), which in all insects examined so far, always belong to the CLIPB family [reviewed in (Kanost and Jiang, 2015)].

ProPO activation cascades have to be strictly regulated to avoid excessive production of harmful byproducts, which could cause systematic damage. Serpins are a superfamily of serine protease inhibitors, which form covalent inhibitory complexes with target proteases (Gettins, 2002; Law et al., 2006; Whisstock and Bottomley, 2006; Meekins et al., 2016). Suppression of proPO activation in insects is mainly achieved through the inhibition of cSPs by a highly conserved serpin, called serpin-3 in *M. sexta* (Zhu et al., 2003), spn27A in *Drosophila melanogaster* (De Gregorio et al., 2002; Ligoxygakis, 2002; Tang et al., 2006), and SRPN2 in *An. gambiae* (Michel et al., 2005; An et al., 2011).

Two principal models of melanization observed in mosquitoes, tissue melanization and humoral melanization of microbes have been used in *An. gambiae* to identify putative CLIPA and B family members of the proPO activation cascade. Tissue melanization, induced by the depletion of SRPN2, the principal serpin that regulates proPO activation in *An. gambiae*, results in the formation of melanotic pseudo-tumors in the hemocoel in the absence of foreign objects or microbial infection (Michel et al., 2005; Michel et al., 2006; An et al., 2011). Humoral melanization, characterized by the deposition of melanin on foreign surfaces, can be induced through implantation of Sephadex beads or the injection of bacteria and fungal spores into the thorax of adult *An. gambiae* (Chun et al., 1995; Schnitger et al., 2007; Yassine et al., 2012b). In addition, humoral melanization of ookinete stages of rodent malaria parasites is induced through knockdown of the c-type lectins CTL4 and CTLMA2 in *An. gambiae* strains that are otherwise susceptible to these parasites (Osta et al., 2004). Targeted RNAi screens in these two models of melanization coupled with biochemical studies revealed a complex CLIP network in *An. gambiae*, which not only entails proteolytic activation among CLIPBs (An et al., 2011; Zhang et al., 2016), but also hierarchical interactions between CLIPAs (Yassine et al., 2014; Nakhleh et al., 2017a; El Moussawi et al., 2019). In addition, the list of CLIPs involved in humoral melanization is dependent on the genetic background as well as the target of melanization (Paskewitz et al., 2006; Volz et al., 2006). Studies in the yellow fever mosquito *Aedes aegypti* have also suggested that humoral and tissue melanization are regulated by distinct proPO activation pathways (Zou et al., 2010). Further complexity of the system can arise through the existence of several PAPs that may act in parallel, as suggested through biochemical analysis of the proPO activation cascades in the model lepidopteran *Manduca sexta* (Jiang et al., 1998; Jiang et al., 2003a; Jiang et al., 2003b; Zhu et al., 2003). Whether parallel proPO activation cascades exist and are invoked separately in tissue and humoral melanization in *An. gambiae* is currently unknown.

To address these open questions, this study focused on the analysis of *An. gambiae* CLIPB10 (Vectorbase Id in AGAMP4.4: AGAP029770, NCBI protein ID: XM_312744.4). CLIPB10 is the closest paralog to CLIPB9, the only bona fide PAP known in mosquitoes (An et al., 2011). CLIPB9 and B10, together with CLIPB8 are physically clustered in a 10 Kb region on chromosome 2R, and are the products of two consecutive duplications of their ancestral gene (Waterhouse et al., 2007; An et al., 2011). In addition, CLIPB10 was shown previously to contribute to humoral melanization of Sephadex beads (Paskewitz et al., 2006). Here, we investigate whether CLIPB10 also contributes to the humoral melanization of rodent malaria parasites as well as tissue melanization. In addition, we explore its enzymatic interactions with its closest CLIPB paralogs and the regulatory effector SRPN2, thus pinpointing CLIPB10 location in the proPO activation cascade.

## Materials and Methods

### Mosquito Strain and Maintenance

The *An. gambiae* G3 strain (MRA-112) was obtained through the MR4 Anopheles Program at the CDC in 2007, and has since then be maintained in the Michel laboratory as described previously (An et al., 2011). Heparinized horse blood (Plasvacc, Templeton, CA, USA) was provided through an artificial membrane feeding system.

### RNAi Experiments Performed in Adult Female Mosquitoes

DsRNA were synthesized as described previously using primers listed in **Table S1** (Michel et al., 2005). One to three day old female were injected with 138 nl of 1.5 µg/µl for each dsRNA. For single knockdown (kd) and double kd controls, ds*GFP* was added to keep the total dsRNA dose constant at 207 ng/mosquito between treatment and controls, as described previously (Zhang et al., 2016).

### Ethics Statement and *Plasmodium berghei* Parasite Infections

This study was carried out in accordance with the recommendations in the Guide for the Care and Use of Laboratory Animals of the National Institutes of Health (Bethesda, USA). Animal protocol was approved by the Institutional Animal Care and Use committee IACUC of the American University of Beirut (permit number 17-10-451). The IACUC functions in compliance with the Public Health Service Policy on the Humane Care and Use of Laboratory Animals (USA), and adopts the Guide for the Care and Use of Laboratory Animals of the National Institutes of Health. *P. berghei* (strain PbGFPCON) constitutively expressing GFP was propagated in BALB/c mice (Franke-Fayard et al., 2004). Mosquitoes were allowed to feed on 5- to 6-week-old anesthetized mice containing a blood parasitemia of 4%–6% for 20 min at 20°C. Mosquitoes were then maintained on 10% sucrose solution at 20°C with a 12-h day-night cycle. Dissection and fixation of mosquito midguts was performed 7 days post blood feeding, followed by counting of fluorescent oocysts and melanized ookinetes with Zeiss fluorescence microscope as previously described (Volz et al., 2006). Five independent biological replicates with at least 42 mosquitoes each were examined.

### Melanotic Tumor Phenotype Assessment in Adult Mosquitoes

DsRNA-injected mosquitoes were generated as described in 2.2 above, and maintained on sugar water for 21 days. To quantify melanotic pseudotumors, abdominal wall of each mosquito was dissected and examined under 40× magnification with Axio Imager A1 microscope (Zeiss) equipped with AxioCamMR5 (Zeiss). Image J was used to quantify the melanized area per abdomen. Two independent biological replicates with 40 mosquitoes each were used.

### Reverse Transcription Quantitative PCR

Efficiency of gene kd was measured by reverse transcription quantitative PCR (RT-qPCR). Briefly, total RNA was isolated from mosquitoes at day 4 post dsRNA injection using TRIzol reagent (Invitrogen). Total RNA (100 ng) was used as the template to synthesize cDNA using iScript cDNA synthesis kit (Bio-Rad) according to the manufacturer’s instructions. RT-qPCR was set up by mixing 0.5 µl cDNA, 0.8 µM primers, and iQ SYBR Green Supermix, followed by amplification on ABI StepOnePlus system (Applied Biosystems). Relative expression of genes of interest was calculated by ΔΔCt method using AgRPS7 as internal reference gene. Three technical replicates were measured for each sample and primer pair.

### Recombinant Serine Protease Expression and Purification

The coding region of proCLIPB10 was amplified by PCR from *An. gambiae* adult cDNA using primers listed in **Table S1**. The forward primer contains a *Not*I restriction site, and the reverse primer includes codons for six histidines followed by a stop codon and a *Hin*dIII restriction site. The PCR product was digested with *Not*I and *Hin*dIII and cloned into the same sites of the expression vector pFastBacI (Invitrogen). The resulting expression vector was used as the template to produce the mutant proCLIPB10_Xa_ expression plasmid following the instructions of QuikChange Multi Site-Directed Mutagenesis Kit (Agilent). CLIPB10 activation site LADR was replaced by IEGR to allow the cleavage and activation by Factor Xa (New England Biolabs). Recombinant constructs were transfected to Sf9 cells using Bac-to-Bac system (Invitrogen), followed by generation of recombinant baculovirus. To express proCLIPB10_Xa_, 800 ml of Sf9 cells (2×10^6^ cells/ml) were infected with the recombinant baculovirus at a multiplicity of infection of 1 and cultured at 27°C with shaking at 140 rpm for 4 days. The medium containing secreted proCLIPB10_Xa_ was harvested by centrifugation at 4°C, 500 × g for 20 min. Na_2_HPO_4_ was added to the 800 ml of cell-free medium to a final concentration of 10 mM, followed by dialysis using 40 mm-wide regenerated cellulose dialysis tubing (Fisher Scientific) against 4 L of 20 mM Na_2_HPO_4_, pH 8.0 thrice for 12 h each at 4°C. The dialyzed medium was supplemented with 50 mM Na_2_HPO_4_, 300 mM NaCl, and 10 mM imidazole, followed by Ni-NTA chromatography (Qiagen) according to manufacturer’s instruction. All 10 ml elution fractions that contained proCLIPB10_Xa_ were pooled and dialyzed using 40 mm-wide regenerated cellulose dialysis tubing against 2 L of 20 mM Tris, 20 mM NaCl, pH 8.0 twice at 4°C. Further purification was performed with Q Sepharose column according to manufacturer’s protocol (GE Healthcare). Fractions containing proCLIPB10_Xa_ were stored at −80°C for future use.

Recombinant proCLIPB8, proCLIPB9, proCLIPB8_Xa_, proCLIPB9_Xa_ and SRPN2 were expressed and purified as described previously (An et al., 2011; Zhang et al., 2016).

### Activation of Recombinant Zymogens

To activate recombinant CLIPB9_Xa_ and CLIPB10_Xa_, 2.5 µg of each purified zymogen was incubated with 1 µg of bovine Factor Xa (New England Biolabs) in a total volume of 50 µl in reaction buffer (20 mM Tris, 100 mM NaCl, 2 mM CaCl_2_, pH 8.0) at 37°C overnight. Two negative controls were set up in parallel, in which either Factor Xa or the zymogen was replaced with same volume of buffer. Cleavage of the zymogen was examined by loading 8 µl of the activation reaction to 10% SDS-PAGE followed by Coomassie blue staining for visualization.

### Substrate Screening of Active CLIPB10_Xa_

To identify a suitable commercial substrate to measure CLIPB10_Xa_ amidase activity, the following chromogenic peptides were tested: N-benzoyl-Ile-Glu-Ala-Arg-*p*-nitroanilide (IEAR*p*Na), N-benzoyl-Phe-Val-Arg-*p*-nitroanilide (FVR*p*Na), N-benzoyl-Pro-Phe-Arg-*p*-nitroanilide (PFR*p*Na), N-benzoyl-Asn-Asn-Asp-Arg-*p*-nitroanilide (NNDR*p*Na), N-benzoyl-Ile-Glu-Gly-Arg-*p*-nitroanilide (IEGR*p*Na), N-benzoyl-Ala-Ala-Pro-Phe-*p*-nitroanilide (AAPF*p*Na), N-benzoyl-Ile-Ala-Gln-Arg-*p*-nitroanilide (IAQR*p*Na), N-benzoyl-Val-Gly-Asn-Lys-*p*-nitroanilide (VGNK*p*Na). CLIPB10_Xa_ was activated as described in *Reverse Transcription Quantitative PCR*, and 3 µl of the activation reaction was added to 200 µl of assay buffer (0.1 M Tris, 0.1 M NaCl, 5 mM CaCl_2_, pH 8.0) containing 500 µM synthetic substrate. Amidase activity was measured by the change of absorbance at 405 nm over 20 min at room temperature. One unit was defined as ΔA_405_ = 0.001/min. A baseline control was set up by measuring the amidase activity of Factor Xa in the absence of proCLIPB10_Xa_ with different substrates. Activity of CLIPB10_Xa_ was calculated by subtracting Factor Xa activity from activated CLIPB10_Xa_ in the presence of Factor Xa. All measurements were performed in two independent replicates.

### Protease-Serpin Complex Formation and MALDI-TOF MS Analysis

Recombinant proCLIPB10_Xa_ was activated by Factor Xa as described above in 2.5, and 20 µl of the activation reaction was incubated with 2 µl of 5 µg/µl purified recombinant SRPN2 (rSRPN2) at room temperature for 1.5 h. Formation of the protease-serpin complex was visualized by 10% SDS-PAGE stained with Coomassie blue. Two bands at ~72 and ~55 kDa were excised from the gel and subjected to in-gel trypsin digestion and Electrospray ionization mass spectrometry analysis (Bruker Daltonics HCT Ultra) at the Biotechnology/Proteomics Core Facility, Kansas State University. Mass spectra were analyzed using Scaffold (4.10.0).

### Inhibition of CLIPB10_Xa_ by rSRPN2

To explore the stoichiometry of SRPN2 inhibition of CLIPB10, purified rSRPN2 was incubated with 0.1 µg of *in vitro* activated CLIPB10_Xa_ at molar ratios of 0, 0.125, 0.25, 0.75, and 1.25, respectively, in the presence of 1 µl BSA (2 µg/µl) at room temperature for 20 min in 20 mM Tris, 100 mM NaCl, pH 8.0. Amidase activity was determined in the presence of 500 µM IEAR*p*Na as described in 2.5 above. Amidase activity at 0:1 (rSRPN2: CLIPB10_Xa_) was defined as 100%. All assays were performed in triplicate.

### Assessment of CLIPB8 and B9 Cleavage by Recombinant CLIPB10_Xa_

CLIPB10_Xa_ was activated by incubating 2.5 µg of purified zymogen with 2.0 µg of bovine Factor Xa (New England Biolabs) in a total volume of 50 µl in reaction buffer (20 mM Tris, 100 mM NaCl, 2 mM CaCl_2_, pH 8.0) at 37°C overnight. Two negative controls were set up in parallel, in which either Factor Xa or the zymogen was replaced with same volume of buffer. Two microliters of the activation reaction was then incubated with 0.8 ul of 126 ng/ul of recombinant purified proCLIPB8 and 0.6 ul of 168 ng/ul of recombinant purified proCLIPB9 in 20 mM Tris, 150 mM NaCl, pH 8.0, respectively, at room temperature for 10 min. Potential cleavage of the CLIPB8 and CLIPB9 zymogens by activated CLIPB10_Xa_, respectively was examined by Western blot. The reactions were treated with 6 × SDS loading buffer (supplemented with β-mercaptoethanol), and heated at 95°C for 5 min. Proteins were separated on 12% SDS-PAGE, and transferred onto a nitrocellulose membrane. Membranes were blocked with 5% milk, and incubated with mouse anti-His antibody (1:2,000) as primary antibody and 1:2,000 diluted goat anti-mouse IgG AP-conjugated secondary antibody (Promega). Alternatively, membranes were incubated with rabbit anti-CLIPB8 antibody [(Zhang et al., 2016), 1:500] and rabbit anti-CLIPB9 antibody [(An et al., 2011), 1:500] as primary antibody, respectively, and 1:5,000 diluted goat anti-rabbit IgG AP-conjugated secondary antibody (Promega). All Western blots were visualized by AP conjugate substrate kit (Bio-Rad).

### Activation of proPO in Plasma

CLIPB9_Xa_ or CLIPB10_Xa_ were activated by Factor Xa as described above in *Reverse Transcription Quantitative PCR*. 8 µl of the activation mixture was incubated with 2 µl of 1:10 diluted plasma collected from day-2 fifth instar *M. sexta* larvae at 37°C for 30 min. Samples were treated with 6 × SDS loading buffer (supplemented with β-mercaptoethanol), heated at 95°C for 5 min. Proteins were separated on 10% SDS-PAGE, and transferred onto a PVDF membrane. The membrane was blocked with 5% milk, and incubated with rabbit anti-*M. sexta* PPO (1:2,000) as primary antibody and 1:20,000 diluted goat anti-rabbit IgG AP-conjugated secondary antibody (Promega). The Western blot was visualized by AP conjugate substrate kit (Bio-Rad). An 8-µl volume of the activation mixture was also used to measure PO activity by adding dopamine to a 2mM final concentration in 50 mM sodium phosphate, pH 6.5. One unit of PO activity was defined as ΔA_470_ = 0.001/min. Three technical replicates were performed.

### Cleavage of Purified proPO *In Vitro*

CLIPB9_Xa_ or CLIPB10_Xa_ were activated by Factor Xa as described above in *Reverse Transcription Quantitative PCR*, and 5 µl of activation mixture was incubated with 0.6 µl of 100 ng/µl purified *M. sexta* proPO (kindly provided by Maureen Gorman, Kansas State University) at 37°C for 30 min. Samples were then subjected to immunoblotting as described above in 2.10.

### Statistical Analyses

Statistical analyses were executed using GraphPad Prism 6.07 Software (GrapPad Software Inc.). Melanotic tumor formation data were evaluated for normality of distribution using Shapiro-Wilk normality test; data were analyzed using (i) Mann Whitney U-test, if comparing two treatment groups, or (ii) Kruskal Wallis test for multiple treatment groups, with Dunn’s Multiple Comparison post-test (*P* < 0.05). Parasite infection data were analyzed by evaluating potential differences in prevalence and infection intensity separately for (i) live oocyst numbers and (ii) melanized ookinetes. Potential differences in prevalence were analyzed using the *χ*^2^ test, by comparing each treatment group to the *CTL4/B10* treatment, followed by Bonferroni correction (*P* < 0.05). Live oocyst and melanized ookinete infection intensity data, respectively were evaluated for normality of distribution using Shapiro-Wilk normality test. Potential differences in infection intensity were analyzed using Kruskal Wallis test for multiple treatment groups, with Dunn’s Multiple Comparison post-test (*P* < 0.05). All enzymatic activity data were evaluated using One-Way ANOVA, with Newman-Keuls post-test (*P* < 0.05).

## Results

### CLIPB10 Is Required for Tissue and *Plasmodium* Melanization

To test the involvement of CLIPB10 in *An. gambiae* tissue melanization, we synthesized dsRNA to perform single and double kd of *SRPN2* and *CLIPB10*. The corresponding dsRNA was injected into 1-2 day female adults, and RT-qPCR was used to test the knockdown efficiency 4 days post injection (**Figure S1**). Transcription level of *CLIPB10* was reduced by over 95% after ds*CLIPB10* or *dsSRPN2/dsCLIPB10* injection. SRPN2 expression level was reduced by 50%, which is similar to previously reported values (Michel et al., 2005; An et al., 2011; Zhang et al., 2016).

**Figure 1:**
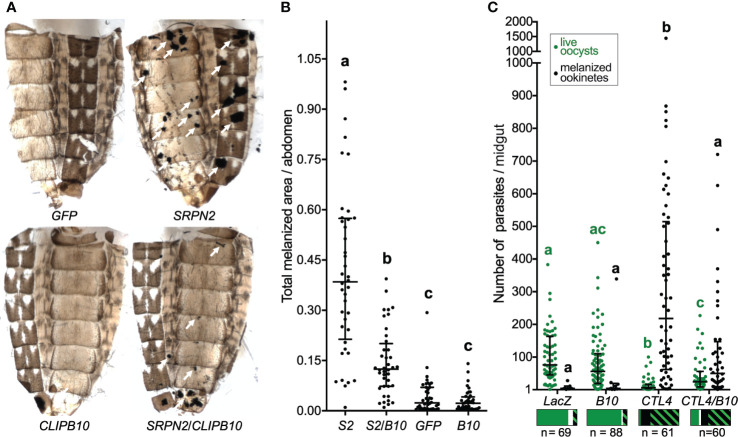
*CLIPB10* knockdown partially reverts ds*SRPN2*- and ds*CTL4*-induced phenotypes. **(A)** Abdominal images were collected 21 days post dsRNA injection. Melanotic tumors are indicated by white arrows. **(B)** Total melanotic areas per abdomen (arbitrary unit) were quantified by Image J. Median and interquartile range are marked (n = 40). Kruskal-Wallis test followed by Dunn’s multiple comparison test were performed to calculate statistical significance. Medians with a common letter are not significantly different (*P* > 0.05). **(C)** Live GFP-expressing oocysts (green circles) and melanized ookinetes (black circles) per gut were scored. Median and interquartile range are marked (n, number of guts examined per treatment group). Both oocyst and melanized ookinete infection intensities, respectively were statistically significantly different between all treatment groups (Kruskal-Wallis test *P* < 0.0001). Medians with a common letter are not significantly different (Dunn’s multiple comparison test, *P* > 0.05), with letters in green indicating live oocyst comparisons, and letters in black indicating melanized ookinetes (see **Table S3** for complete summary statistics). The bar charts below each treatment group show prevalence as the fraction of guts containing live oocysts only (green), melanized ookinetes only (black), both live oocysts and melanized ookinetes (green/black hatch), and no parasites (white). The statistical analyses of the prevalence data are summarized in **Table S4**.

The kd of SRPN2 caused severe melanotic tumors on the abdomen of mosquitoes, which were partially reversed by *CLIPB10* kd in SRPN2-depleted mosquitoes (**Figure 1A**). Quantification of melanotic areas per abdomen demonstrated a significant reduction in melanization in ds*SRPN2*/ds*CLIPB10* as compared to ds*SRPN2*-treated mosquitoes (**Figure 1B**). Neither ds*GFP* nor ds*CLIPB10* injection alone caused recognizable melanotic tumors in mosquitoes.

To test whether CLIPB10 is also required for melanization of malaria parasites, adult female mosquitoes were injected with ds*CTL4* and ds*CLIPB10* to induce gene silencing, and subsequently given an infectious blood meal containing GFP-expressing *P. berghei*. Live oocysts with green fluorescence and dead melanized ookinetes were scored in the midguts of dissected mosquitoes at day 7 after blood feeding (**Table S2**). Silencing of *CLIPB10* did not alter parasite development, as the median numbers of live oocysts and melanized ookinetes per gut in ds*CLIPB10* mosquitoes was similar to the numbers observed in the ds*LacZ*-injected control group (**Figure 1C** and **Table S3**). However, in ds*CTL4* mosquitoes, silencing *CLIPB10* tripled the median number of live oocysts, and quartered the median number of melanized ookinetes (**Figure 1C** and **Table S3**). To further investigate the impact of *CLIPB10* kd on parasite development, we determined the prevalence of parasite infection by determining the percentage of mosquitoes whose guts carried live oocysts. In addition, we also analyzed the percentage of mosquitoes whose midguts contained melanized ookinetes (**Table S4** and **Figure 1C**). Co-silencing *CLIPB10* and *CTL4* did not change the prevalence of live oocysts as compared to *CTL4*-silenced mosquitoes (*χ*^2^ test, *P* = 0.2905, **Table S4**), suggesting that CLIPB10 is probably not involved in parasite killing. In contrast, co-silencing *CLIPB10* and *CTL4* significantly reduced the percentage of mosquitoes bearing melanized ookinetes (*χ*^2^ test, *P* = 0.0003, **Table S4**), further supporting the role of CLIPB10 in the humoral melanization of parasites.

### Active Recombinant proCLIPB10_Xa_ Exhibits Amidase Activity

The reverse genetic analysis revealed that CLIPB10 is required for humoral and tissue melanization. To determine whether CLIPB10 regulates melanization by promoting the activation of proPO, we characterized the molecular functions of CLIPB10 using biochemical approaches. The annotated full-length coding sequence of CLIPB10 encodes a 362 amino acid long protein with a predicted 19 amino acid long signal peptide at the N-terminus. The mature protein contains a single, canonical type 2 clip domain signature of C-X_9_-C-X_5_-C-X_26_-C-X_7_-CC at the N-terminus, and a S1A protease domain with the conserved H-D-S catalytic triad at the C-terminus. These two domains are separated by a linker region, which contains the LADR putative activation cleavage site. Since CLIPB10 is expressed as a zymogen, and its endogenous activating protease is currently unknown, we expressed a recombinant, mutated version of the proCLIPB10 that enables its cleavage *in vitro* by commercially available bovine Factor Xa. SDS-PAGE analysis of purified recombinant proCLIPB10_Xa_ reveals a mass of approximately 43 kDa (**Figure S2**), which is slightly higher than the predicted molecular weight of 39.1 kDa. This difference is likely due to glycosylation, as the protein sequence contains two predicted N-linked glycosylation sites. Addition of Factor Xa to purified recombinant proCLIPB10_Xa_ resulted in the appearance of a band around 38 kDa, which matches the predicted size of the catalytic domain of CLIPB10_Xa_, indicating that factor Xa efficiently cleaves proCLIPB10_Xa_. To identify the optimal artificial substrate for measuring the amidase activity of CLIPB10, we screened eight short chromogenic peptides with activated CLIPB10_Xa_. Activated CLIPB10_Xa_ showed amidase activity against peptide substrates with arginine at the P1 site, including IEAR*p*NA (**Figure S3**).

### Active CLIPB10_Xa_ Is Directly Inhibited by Recombinant SRPN2

**Figure 2:**
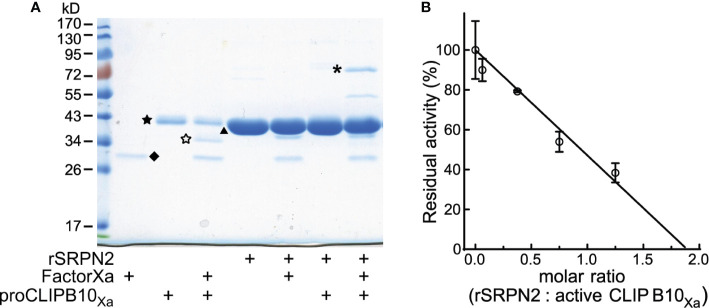
Active CLIPB10_Xa_ is inhibited by rSRPN2. **(A)** SDS-PAGE and Coomassie blue staining shows the presence of a covalent complex formed by rCLIPB10_Xa_ and rSRPN2, indicated by asterisk. CLIPB10_Xa_ zymogen is indicated by a filled star, catalytic domain is indicated by an open star, factor Xa is indicated by diamond, and rSRPN2 is indicated by triangle. **(B)** IEAR*p*Na was used as the substrate to measure the residual amidase activity of active CLIPB10_Xa_, which was inhibited by rSRPN2 at increasing molar ratios. Data are shown as means ± S.D. (n = 3). The stoichiometry of inhibition is 1.89.

Inhibition of proteases by serpins requires the formation of a SDS-stable complex of the serpin and its cognate protease. To determine if CLIPB10 can be inhibited by SRPN2, we first tested whether these two proteins form such complexes *in vitro*. Activated CLIPB10_Xa_ was incubated with purified recombinant SRPN2 and complex formation was analyzed by SDS-PAGE. When SRPN2 was added to activate CLIPB10_Xa_ the 38 kDa band corresponding to the protease domain of CLIPB10 was not detected, and instead, a higher molecular weight band of around 72 kDa was observed, which matched the predicted molecular weight of a CLIPB10_Xa_:rSRPN2 inhibitory complex (**Figure 2A**). Additionally, a second higher molecular weight band of 55 kDa appeared, which likely constitutes a partially degraded form of the inhibitory complex (**Figure 2A**). Analysis of tryptic peptides from both the 72- and 55-kDa bands by ESI-MS identified both SRPN2 and CLIPB10 in both bands, confirming the formation of covalent complexes between CLIPB10_Xa_ and rSRPN2 (**Figure S4**). To confirm that this complex formation indeed leads to inhibition of CLIPB10, we tested the ability of SRPN2 to inhibit the IEARase activity of activated CLIPB10_Xa_ *in vitro*. CLIPB10 activity decreased linearly with increasing concentrations of SRPN2 (**Figure 2B**). The stoichiometry of inhibition was 1.89, indicating that, under the used experimental conditions, SRPN2 acts as an inhibitor and not as substrate for CLIPB10.

### CLIPB10_Xa_ Promotes proPO Cleavage and Activation in *Manduca sexta* Plasma

**Figure 3:**
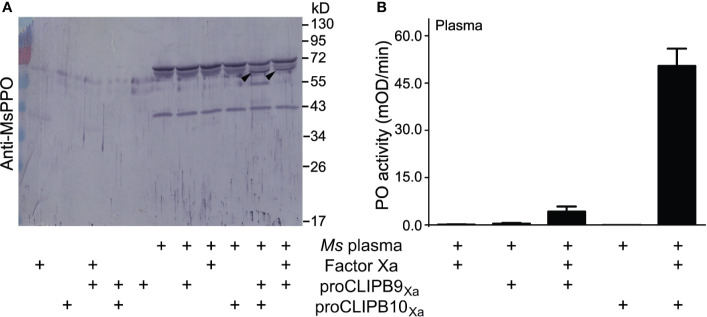
Plasma proPO is cleaved and activated by active rCLIPB10_Xa_. **(A)** Purified recombinant proCLIPB9_Xa_ or proCLIPB10_Xa_ were activated by Factor Xa, and was added individually *to M. sexta* plasma. The Western blot of the reactions was probed with anti-*M. sexta* PPO antibody. Black arrows indicate the band that correspond to *M. sexta* PO. **(B)** PO activity was observed when plasma was supplemented with activated CLIPB9_Xa_ or CLIPB10_Xa_. Data are shown as means ± S.D (n = 6).

The biochemical analysis of proPO activation in mosquitoes is hindered by the limited amount of hemolymph that can be extracted. This shortage can be overcome by using the *M. sexta* model system as a source of proPO (Michel et al., 2006; An et al., 2011; Zhang et al., 2016). We used plasma from *M. sexta* larvae to explore the impact of CLIPB10 on the proPO activation cascade. *M. sexta* plasma samples were pre-screened as previously described (Tong and Kanost, 2005). Activated CLIPB10_Xa_ was incubated with *M. sexta* plasma, followed by western blot analysis using anti-*M. sexta* PO antibody. A doublet band around 80 kDa in *M. sexta* plasma represents heterodimeric proPO consisting of 79-kDa proPO1 and 80-kDa proPO2 (Jiang et al., 1997). Addition of activated CLIPB10_Xa_ to *M. sexta* plasma resulted in the appearance of a 70-kDa doublet band corresponding to *M. sexta* active PO (**Figure 3A**), and an additional band around 55kD of unknown identity. The same doublet band was observed in the plasma supplemented with activated CLIPB9_Xa_, which we identified previously as a functional PAP in *An. gambiae* (An et al., 2011). PO activity of plasma increased in the presence of active CLIPB9_Xa_, and increased even more in the presence of active CLIPB10_Xa_ (**Figure 3B**). These results confirm that the proteolytic activity of CLIPB10 promotes proPO cleavage and PO activity.

### CLIPB10_Xa_ Functions as a Prophenoloxidase Activating Protein

**Figure 4:**
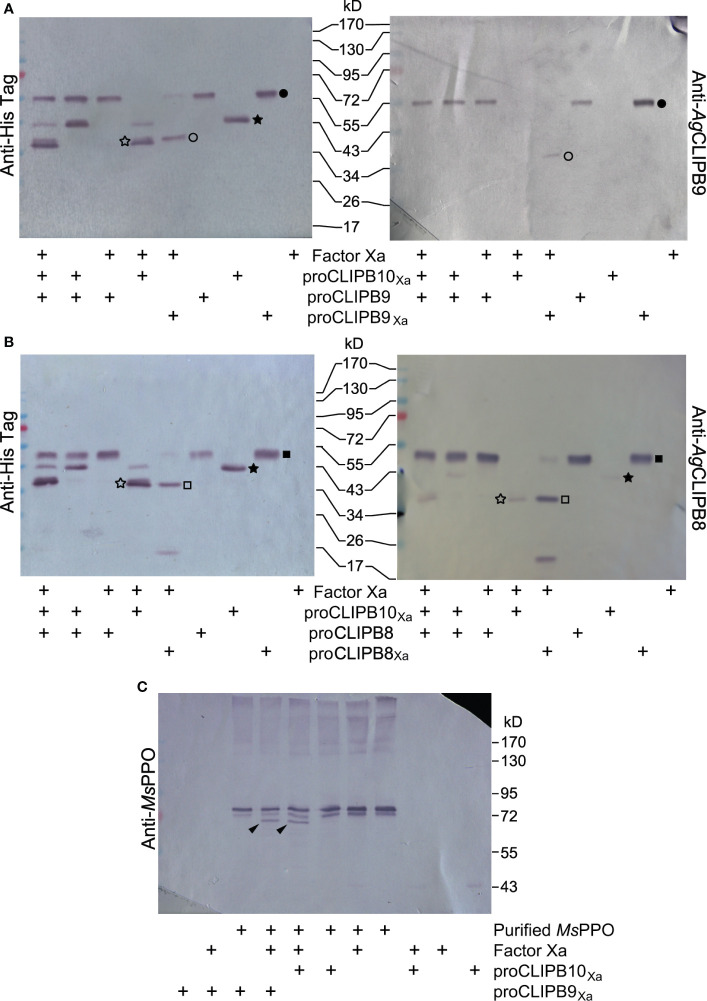
Placement of CLIPB10 in the proPO activation cascade. **(A)** Neither activated rCLIPB10_Xa_, nor commercial Factor Xa cleave recombinant wild-type proCLIPB9. **(B)** Neither activated rCLIPB10_Xa_, nor commercial Factor Xa cleave recombinant wild-type proCLIPB8. All recombinant proteins contain a His-tag at their C-terminus, which is detected by anti-His antibody (left panels), and are also recognized by their specific antibodies. Filled star, proCLIPB10_Xa_; open star, protease domain of rCLIPB10_Xa_; filled circle, zymogen form of the recombinant proCLIPB9 and proCLIPB9_Xa_, open circle, protease domain of rCLIPB9_Xa_; filled square, zymogen form of the recombinant proCLIPB8 and proCLIPB8_Xa_; open square, protease domain of rCLIPB8_Xa_. **(C)** Activated rCLIPB10_Xa_ directly cleaves *M. sexta* proPO. Black arrows indicate a smaller PO band on Western blots probed with anti-*M. sexta* PPO antibody.

To determine the placement of CLIPB10 in the proPO activation cascade, we used a targeted approach to identify nascent protein substrates of CLIPB10 using recombinant proteins. We first tested whether CLIPB10 promotes PO activity by activating proCLIPB9, the only known terminal protease of the proPO activation cascade in *An. gambiae*. To test whether CLIPB10 can cleave proCLIPB9 *in vitro*, active rCLIPB10_Xa_ was incubated with recombinant proCLIPB9, followed by western blot analysis using either anti-His antibody or anti-CLIPB9 antibody. Addition of active CLIPB10_Xa_ did not result in the cleavage of proCLIPB9 (**Figure 4A**), suggesting that CLIPB10 is not directly upstream of CLIPB9. Addition of active CLIPB8_Xa_ also did not lead to cleavage of proCLIPB9, as reported previously (Zhang et al., 2016).

We next tested whether CLIPB10 promotes PO activity by activating proCLIPB8, a protease required for tissue melanization, which is upstream of CLIPB9 in the proPO activation cascade in *An. gambiae*. To test whether CLIPB10 can cleave proCLIPB8 *in vitro*, active rCLIPB10_Xa_ was incubated with recombinant proCLIPB8, followed by western blot analysis using either anti-His antibody or anti-CLIPB9 antibody. Addition of active CLIPB10_Xa_ did not result in the cleavage of proCLIPB9 (**Figure 4B**), suggesting that CLIPB10 is not directly upstream of CLIPB9.

To explore whether CLIPB10 directly catalyzes proPO activation, active CLIPB10_Xa_ was incubated with purified *M. sexta* proPO, followed by western blot analysis using anti-*M. sexta* PO antibody. Heterodimeric *M. sexta* proPO remained intact after incubation with Factor Xa, proCLIPB10_Xa_, and proCLIPB9_Xa_, respectively. In contrast, both active CLIPB10_Xa_ and CLIPB9_Xa_ were able to cleave *M. sexta* proPO *in vitro*, as demonstrated by the appearance of a doublet band corresponding to *M. sexta* PO (**Figure 4C**). These data demonstrate that CLIPB10, similar to CLIPB9, can function as a PAP in insect hemolymph.

## Discussion

To investigate the complexity of immune regulation in *An. gambiae*, this study examined the molecular make-up of the protease cascades that control melanization *via* proteolytic proPO activation. Specifically, this study set out to answer the following two questions: Firstly, do parallel proPO activation cascades exist in *An. gambiae*, as they do in other insect species, including *M. sexta* (Jiang et al., 1998; Jiang et al., 2003a; Jiang et al., 2003b). Secondly, are separate proPO activation cascades involved in tissue and humoral melanization as suggested in *Ae. aegypti* (Zou et al., 2010). To address these questions, we focused our analysis on CLIPB10, based on the results of previous targeted reverse genetic screens and phylogenetic analyses of *CLIPB*s performed by us and others (Paskewitz et al., 2006; An et al., 2011; Cao et al., 2017).

To answer the first question, we examined the molecular function of CLIPB10 by biochemical means. The hallmark of parallel proPO activation cascades is the existence of two or more PAPs that independently cleave and activate proPO, and cross-talk between cascades is observed at levels upstream of the terminal PAP (e.g., An et al., 2009; Wang Y. et al., 2020). We posited a role of CLIPB10 as a PAP based on the following existing data. Previously, we had identified the first PAP, CLIPB9 (AGAP029769) and its inhibitor, SRPN2 (AGAP006911) in *An. gambiae* (An et al., 2011). Our analysis of the phylogenetic relationships among *An. gambiae* CLIPB proteases clustered CLIPB9 and CLIPB10 (AGAP029770) with *M. sexta* PAP1 and *D. melanogaster* MP2, to the exclusion of other *An. gambiae*, *M. sexta* and *D. melanogaster* sequences. Given that CLIPB9, PAP1 and MP2 are known terminal proteases in proPO activation cascades, CLIPB10 was therefore an excellent candidate for PAP function in *An. gambiae* (Jiang et al., 1998; Tang et al., 2006; An et al., 2013). Using recombinant protein expressed with the baculovirus expression system, we obtained active CLIPB10 protein *in vitro*. Activated CLIPB10 added to *M. sexta* plasma results in significant activation cleavage of proPO and PO activity. Activated CLIPB10 also resulted in activation cleavage of purified *M. sexta* proPO. Therefore, CLIPB10 can function as the terminal protease in the proPO activation cascade. Given that seven out of eight proPOs of *An. gambiae* share the same activation cleavage site with *M. sexta* proPO (Jiang et al., 2003a; Michel et al., 2005), the data presented here strongly suggest that CLIPB10 functions as a PAP in the hemolymph of *An. gambiae* mosquitoes.

We previously showed that SPRN2 functions as a master regulator of melanization in mosquitoes, by inhibiting the PAP function of CLIPB9 (An et al., 2011). In this current study, we show that SRPN2 also inhibits activated CLIPB10 *in vitro*, as demonstrated by the formation of inhibitory protease-serpin complexes, and the reduction of CLIPB10’s amidase activity with increasing molar ratios of SRPN2 to CLIPB10 *in vitro*. This interaction of *CLIPB10* and *SRPN2* also occurs *in vivo*. The total area of melanized tumors in the abdomen of *SRPN2* kd mosquitoes was decreased by 65% when *CLIPB10* kd was also invoked. Together, these results demonstrate the existence of two PAPs in *An. gambiae*, and suggests that two proPO activation cascades exist in this mosquito species. This parallels the findings in *M. sexta*, where two separate proPO activation cascades result in the activation of PAP1 and PAP2/PAP3, respectively (Jiang et al., 1998; Jiang et al., 2003a; Jiang et al., 2003b). The activity of terminal PAPs in both proPO activation cascades in *M. sexta* are inhibited by the same serpin, Serpin-3 (Zhu et al., 2003; Christen et al., 2012). We find the same to be true for the two putative proPO activation cascades in *An. gambiae*. SRPN2, the ortholog of *M. sexta* Serpin-3 inhibits both CLIPB9 and CLIPB10 with similar stoichiometry of inhibition (1.3 for SRPN2:CLIPB9, and 1.7 for SRPN2:CLIPB10), indicating both PAPs are subjected to efficient SRPN2 sequestration. Beyond being inhibited by the same serpin, we thus far have found no evidence for additional interactions between the CLIPB9 and B10 PAPs, as recombinant activated CLIPB10 did not activate CLIPB9 *in vitro*. Preliminary triple knockdown analyses suggests additive effects between the two PAPs, as double kd of *CLIPB9* and *B10* further reduced melanotic tumour formation due to SRPN2 depletion (Zhang and Michel, unpublished). However, epistasis analyses using RNAi are limited by the incomplete knockdown of *CLIPB9*, and thus will be explored in future analyses beyond the scope of this current study.

To address the second question of whether separate proPO activation cascades lead to tissue and humoral melanization in *An. gambiae*, we examined the function of CLIPB10 in two distinct models of melanization in *An. gambiae*. The study by Paskewitz *et al*. had implicated CLIPB10 in the humoral melanization of Sephadex beads in the hemocoel of adult female *An. gambiae* (Paskewitz et al., 2006), as *CLIPB10* kd resulted in a small but significant reduction in the number of Sephadex beads that in control mosquitoes were 100% melanized after overnight incubation. Our analysis of the role of CLIPB10 in *CTL4* kd-mediated melanization of rodent *P. berghei* parasites further supports the role of CLIPB10 in humoral melanization. *CLIPB10/CTL4* double*-*kd reduced the average number of melanized ookinetes per midgut by 77% as compared to *CTL4* kd mosquitoes. While *CLIPB10* kd quadrupled the number of live oocysts as compared to CTL4 kd, live oocyst numbers remained one third of those observed in control mosquitoes. Thus, CLIPB10 is largely required for melanin deposition on the parasite surface and to a lesser degree impacts the parasite killing observed in *CTL4* kd mosquitoes. Taken together, these data clearly demonstrate that CLIPB10 is required for humoral melanization of parasites and to a lesser extent Sephadex beads, as well as tissue melanization. This is in contrast to findings in *Ae. aegypti*, were two distinct regulatory modules of tissue melanization and hemolymph proPO activation were described (Zou et al., 2010). Tissue melanization in *Ae. aegypti* requires two cSPs, TMP, the ortholog of *An. gambiae* CLIPB8, and IMP-1, the ortholog of *An. gambiae* CLIPB9, and is inhibited by serpin-2, the ortholog of *An. gambiae* SRPN2. In contrast, humoral melanization, which the authors analyze through the cleavage of proPO in the hemolymph, is inhibited by serpin-1 and serpin-3, and requires the action of IMP-1 as well as IMP-2. In contrast, our results suggest that in *An. gambiae*, proPO activation cleavage is mediated by both CLIPB9 and CLIPB10, and inhibited directly by SRPN2. Our data strongly suggest that the difference between humoral and tissue melanization in *An. gambiae* is not due to the action of different proPO activation cascades. Instead, tissue melanization is the consequence of dysregulated melanogenesis due to constitutively active proPO activation cascades induced by *SRPN2* kd. This dysregulated melanogenesis is also exemplified by the dramatic activation of key regulators of humoral melanization, such as CLIPA8, CLIPA28, and CLIPA14, in the hemolymph of naive *SRPN2* kd mosquitoes (El Moussawi et al., 2019). Melanogenic reactions must occur highly localized on the surface of target entities to prevent adverse impact on self-entities, as unregulated melanogenesis produces harmful byproducts and intermediates that can disperse through hemolymph circulation and result in systemic damage (Nappi and Christensen, 2005). In *SRPN2* kd mosquitoes, this systemic damage is not only visualized by tissue melanization, but can also be measured by concomitant reduced lifespan (Michel et al., 2005; Volz et al., 2005; Volz et al., 2006; An et al., 2011; Zhang et al., 2016). While the localization of melanization on microbial surfaces is achieved by the opsonizing function of TEP1 (Blandin et al., 2004; Yassine et al., 2012a; Povelones et al., 2013), future studies will have to determine whether TEP1 and/or other components of the complement pathway are also required for tissue melanization.

Both CLIPB9 and CLIPB10 are secreted as zymogens into the hemolymph of *An. gambiae*, and require activation cleavage. However, their nascent activating proteases are currently unknown. Studies in other model insects have shown that PAP activation cleavage is mediated by cSPs belonging to the CLIPC family, which in turn are proteolytically activated by an upstream modular serine protease (ModSP). Examples of such proPO activation cascades have been described in several model organisms, including *M. sexta* (Gorman et al., 2007; Wang and Jiang, 2007), *Tenebrio molitor* (Kan et al., 2008; Jiang et al., 2009), and most recently *Helicoverpa armigera* (Wang Q. et al., 2020). While currently no data exist that show activation cleavage of either CLIPB9 or CLIPB10 by an endogenous CLIPC, recent genetic evidence suggests that CLIPC9 is required for humoral melanization of parasites as well as tissue melanization (Sousa et al., 2020). Concomitant knockdown of *CTL4* and *CLIPC9* reversed ookinete melanization without rescuing parasite killing, thus phenocopying the *CTL4*/*CLIPB10* double-kd. CLIPC9 is thus an excellent candidate for a PAP-activating protease in *An. gambiae*, and a potential activator of CLIPB10 during infection-induced melanization. The data provided by Sousa and co-authors also suggest that CLIPC9 undergoes localized activation cleavage, binding to microbial surfaces in a cleaved form (Sousa et al., 2020). The cleavage of CLIPC9 depends on components of the complement-like pathway, but the nascent protease mediating CLIPC9 cleavage awaits identification. A ModSP, which potentially could function as a CLIPC9-activating protease is SP217. SP217 has a similar domain structure to *M. sexta* HP14, the only known ModSP to function in proPO activation upstream of PAP1/PAP2 (Wang and Jiang, 2007). Addition of recombinant SP217 to *M. sexta* plasma increased PO activity, suggesting that SP217 may substitute HP14 function (Wang Y. et al., 2020). This notion is further supported by our finding that *SP217* kd significantly reduced *SRPN2* kd-mediated melanotic tumor formation (Zhang and Michel, unpublished). Future studies will have to determine whether SP217, CLIPC9, and CLIPB9 or B10 indeed constitute proPO activation cascades in *An. gambiae*.

In summary, our study demonstrates that CLIPB10 is the second prophenoloxidase-activating enzyme identified in *An. gambiae*, in addition to the previously identified CLIPB9, suggesting functional redundancy in the cSP network that controls melanization. In addition, our data suggest that tissue melanization and humoral melanization are at least partially mediated by the same CLIPB proteases. Studies are currently underway to determine whether additional PAPs exist, and in how far these parallel proPO activation cascades provide true functional redundancy or are evoked non-redundantly dependent on the immune challenge.

## Data Availability Statement

The original contributions presented in the study are included in the article/**Supplementary Materials**. Further inquiries can be directed to the corresponding author.

## Ethics Statement

The animal study was reviewed and approved by the Institutional Animal Care and Use committee IACUC of the American University of Beirut.

## Author Contributions

XZ, LM, SS, and SZ performed the experiments and analyzed the data. KM and ML drafted the manuscript. XZ, MO, and KM designed the experiments. All authors contributed to the article and approved the submitted version.

## Funding

This study was supported by funding from the National Institutes of Health grant numbers R01AI095842 and R01AI140760, and the USDA National Institute of Food and Agriculture, Hatch project 1021223 to KM. This is contribution no. 21-011-J from the Kansas Agricultural Experiment Station. Its contents are solely the responsibility of the authors and do not necessarily represent the official views of the funding agencies.

## Conflict of Interest

The authors declare that the research was conducted in the absence of any commercial or financial relationships that could be construed as a potential conflict of interest.
