# Supplementary material for: CLIPB10 is a Terminal Protease in the Regulatory Network That Controls Melanization in the African Malaria Mosquito Anopheles gambiae
Source: Front Cell Infect Microbiol. 2021 Jan 15;10:585986. doi: 10.3389/fcimb.2020.585986 (PMC7843523; doi:10.3389/fcimb.2020.585986)
Supplement: Supplementary file 1 [file Image_1.pdf]

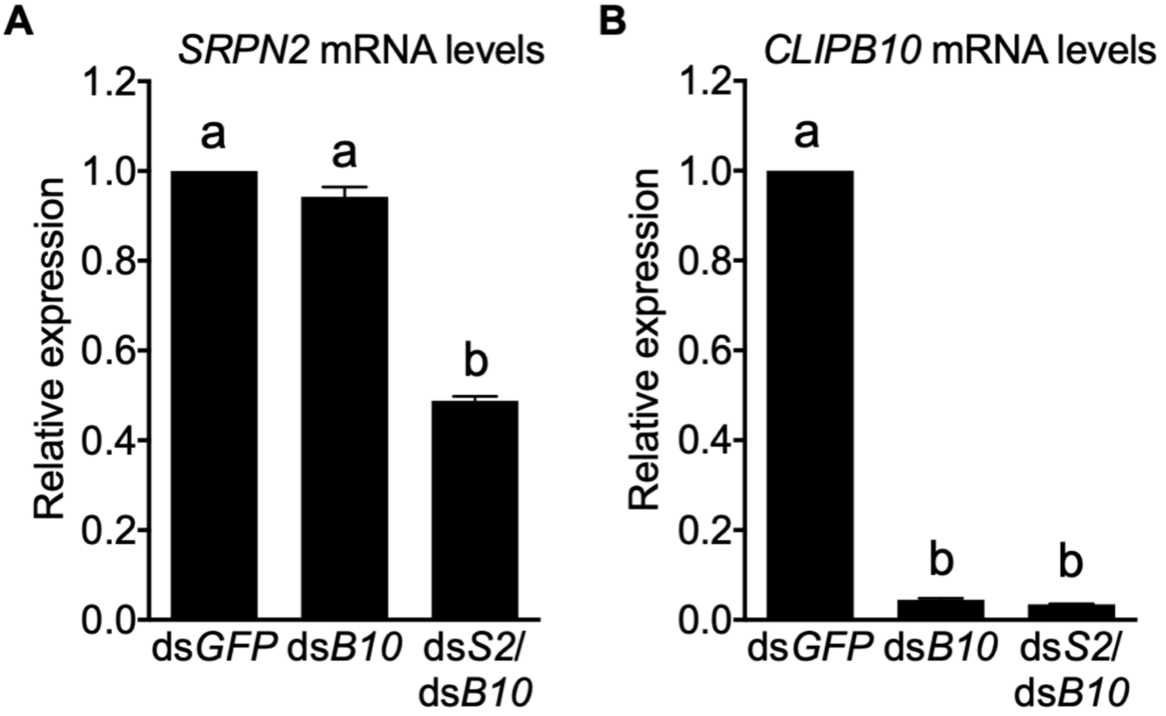

Figure S1. Gene knockdown efficiency. RT-qPCR was used to measure the relative expression levels with ribosomal protein S7 as the internal reference and ds*GFP*-treated samples as the calibrator. Relative mRNA levels of SRPN2 (**A**) and CLIPB10 (**B**) are shown as means ± SEM (n=3). Results of statistical analysis (one-way ANOVA followed by Newman-Keuls test, $P < 0.05$) are indicated. Means with the same letter are not significantly different.
