# Supplementary material for: CLIPB10 is a Terminal Protease in the Regulatory Network That Controls Melanization in the African Malaria Mosquito Anopheles gambiae
Source: Front Cell Infect Microbiol. 2021 Jan 15;10:585986. doi: 10.3389/fcimb.2020.585986 (PMC7843523; doi:10.3389/fcimb.2020.585986)
Supplement: Supplementary file 2 [file Image_2.pdf]

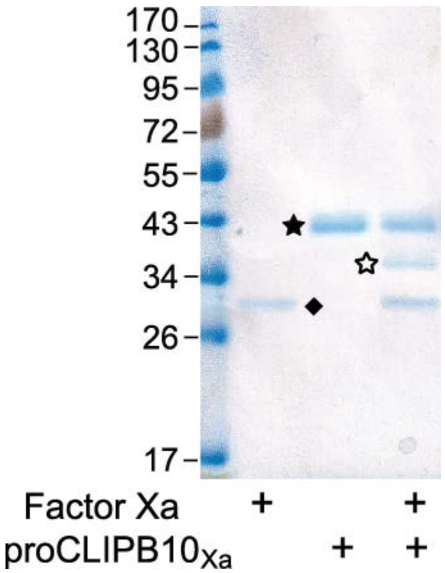

Figure S2. Activation of recombinant proCLIPB10$_{Xa}$ by factor Xa. Successful cleavage of proCLIPB10$_{Xa}$ at IEAR activation site by factor Xa was confirmed by SDS-PAGE (reducing) and Coomassie blue staining. Diamond, factor Xa; black star, proCLIPB10$_{Xa}$ zymogen; open star, CLIPB10$_{Xa}$ catalytic domain.
