# Supplementary material for: CLIPB10 is a Terminal Protease in the Regulatory Network That Controls Melanization in the African Malaria Mosquito Anopheles gambiae
Source: Front Cell Infect Microbiol. 2021 Jan 15;10:585986. doi: 10.3389/fcimb.2020.585986 (PMC7843523; doi:10.3389/fcimb.2020.585986)
Supplement: Supplementary file 3 [file Image_3.pdf]

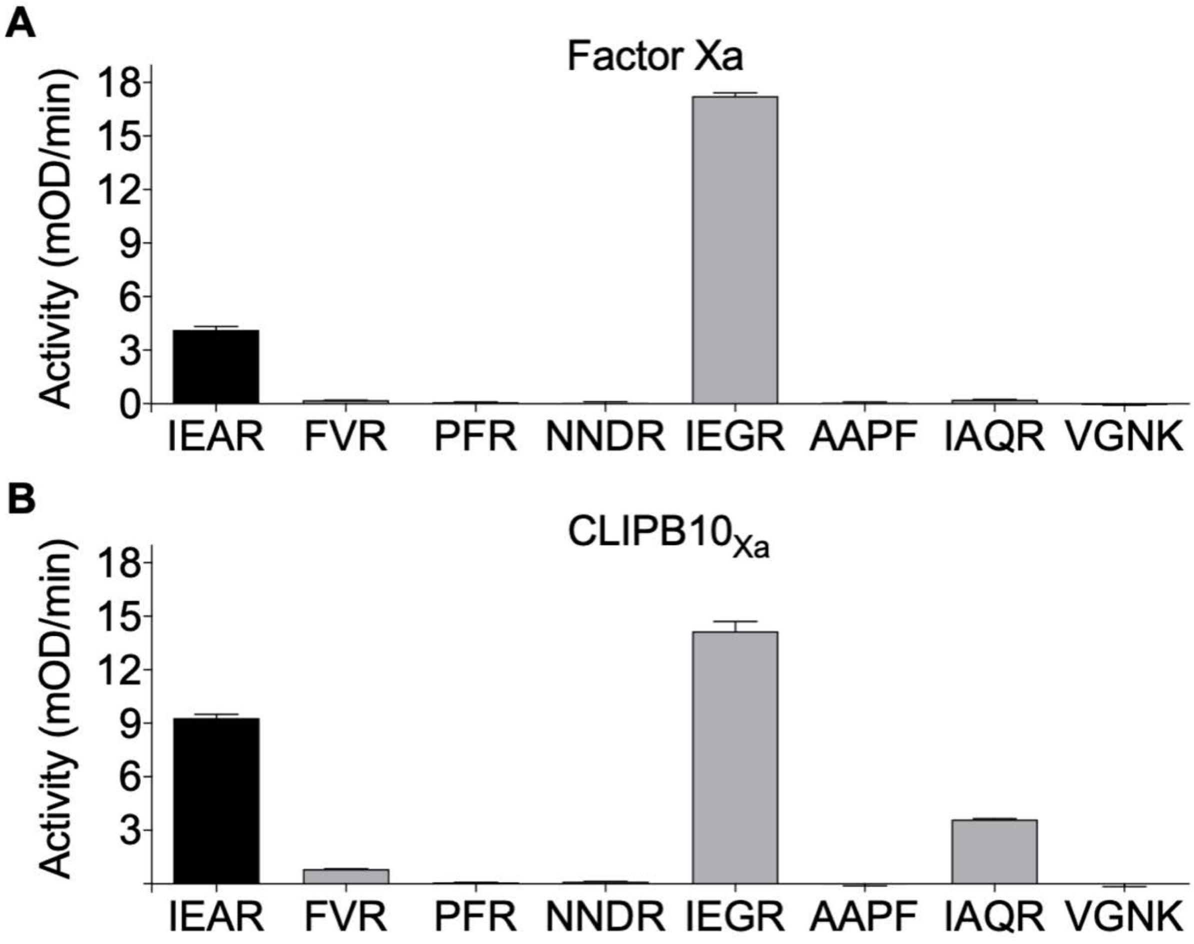

Figure S3. Substrate screening of active CLIPB10$_{Xa}$. Commercially available substrates linked with chromogenic group, *p*-nitroanilide, were used to determine amidase activity of CLIPB10$_{Xa}$. N-benzoyl-Ile-Glu-Ala-Arg-*p*-nitroanilide (IEAR*p*Na), N-benzoyl-Phe-Val-Arg-*p*-nitroanilide (FVR*p*Na), N-benzoyl-Pro-Phe-Arg-*p*-nitroanilide (PFR*p*Na), N-benzoyl-Asn-Asn-Asp-Arg-*p*-nitroanilide (NNDR*p*Na), N-benzoyl-Ile-Glu-Gly-Arg-*p*-nitroanilide (IEGR*p*Na), N-benzoyl-Ala-Ala-Pro-Phe-*p*-nitroanilide (AAPF*p*Na), N-benzoyl-Ile-Ala-Gln-Arg-*p*-nitroanilide (IAQR*p*Na), N-benzoyl-Val-Gly-Asn-Lys-*p*-nitroanilide (VGNK*p*Na). (**A**) Amidase activities of Factor Xa were measured with substrates. (**B**) Activity of CLIPB10Xa was calculated by subtracting factor Xa activity from activated CLIPB10Xa in the presence of factor Xa. IEAR is the most suitable substrate for CLIPB10$_{Xa}$. Data are shown as means ± S.D. (n=2).
