# Supplementary material for: CLIPB10 is a Terminal Protease in the Regulatory Network That Controls Melanization in the African Malaria Mosquito Anopheles gambiae
Source: Front Cell Infect Microbiol. 2021 Jan 15;10:585986. doi: 10.3389/fcimb.2020.585986 (PMC7843523; doi:10.3389/fcimb.2020.585986)
Supplement: Supplementary file 4 [file Image_4.pdf]

A

Upper band

SRPN2 (AGAP006911-PA, XP_308845.4)

*MNKLNFVILCLAALLVFDATA*QQDVHGPFQGQRQNEFDL**M**FVKEIFKNHNSNVVLSPFSVKILL
TLIYEASDTSFGNAVSNTKRELSSVIQNDNIDHTRSYYKQLLESAQQDNKDYDLNIATNFFVDD
FIEVINKYQQIANTHYHA**M**LEKVSYSNPTQTAATINNWVSEHTNGRLREIVTPDSLEGAVITLV
NVIYFKGLWTYPFPEVANNVKPFYGTRGKPTNAQY**M**EQNGQFYYDNSADLGAQILRLPYRGNKL
A**M**YFILPNPDNTVNQVLDRINSASLHQALWYMEENEVNVTLPKFKFDFSEQLNEPLQQVGIREI
FSQNASLPLLARGRGARDEVRVSRIFQKAGITINELGSEAYAATEIQLVNKFGGDGVQIFNANR
PFIFFIEDETLGTMLFAGKIENPVF

CLIPB10 (AGAP003058-PA, XP_312744.4)

*MAKVVDCVLLLAFIAVVRG*QEACRTPDHRDGVCHPVQQCPSVRDEFFNSDRVLSEDEIDYLRKL
QCKTKDVTICCPDGVTTVDRNPTAVRDGLPNPKAFECGLDTLADRIIGGNYTAIDEFPWYALLE
YQSKKGERAFKCGGSLINGRYVLTAAHCLANKKLDEGERLVNVRLGEYNTATDTDCADGNPDDC
ADPPQNFGIEAQIVHPGYDKNGPYQHHDIALIRLDRDVTMNNFVSPVCLPPDDFPPTSPGLNVT
AVGFGHTGRQRHSGIKKKAQFPVFAQEECDKKWKNIEVIGEQLCAGGVFGIDSCSGDSGGPL**M**V
KRFYWIQEGVISFGNQCALEGWPGVYTRVSSYLDWIRQNIRR

B

Lower band

SRPN2 (AGAP006911-PA, XP_308845.4)

*MNKLNFVILCLAALLVFDATA*QQDVHGPFQGQRQNEFDL**M**FVKEIFKNHNSNVVLSPFSVKILL
TLIYEASDTSFGNAVSNTKRELSSVIQNDNIDHTRSYYKQLLESAQQDNKDYDLNIATNFFVDD
FIEVINKYQQIANTHYHAMLEKVSYSNPTQTAATINNWVSEHTNGRLREIVTPDSLEGAVITLV
NVIYFKGLWTYPFPEVANNVKPFYGTRGKPTNAQY**M**EQNGQFYYDNSADLGAQILRLPYRGNKL
A**M**YFILPNPDNTVNQVLDRINSASLHQALWYMEENEVNVTLPKFKFDFSEQLNEPLQQVGIREI
FSQNASLPLLARGRGARDEVRVSRIFQKAGITINELGSEAYAATEIQLVNKFGGDGVQIFNANR
PFIFFIEDETLGTMLFAGKIENPVF

CLIPB10 (AGAP003058-PA, XP_312744.4)

*MAKVVDCVLLLAFIAVVRG*QEACRTPDHRDGVCHPVQQCPSVRDEFFNSDRVLSEDEIDYLRKL
QCKTKDVTICCPDGVTTVDRNPTAVRDGLPNPKAFECGLDTLADRIIGGNYTAIDEFPWYALLE
YQSKKGERAFKCGGSLINGRYVLTAAHCLANKKLDEGERLVNVRLGEYNTATDTDCADGNPDDC
ADPPQNFGIEAQIVHPGYDKNGPYQHHDIALIRLDRDVTMNNFVSPVCLPPDDFPPTSPGLNVT
AVGFGHTGRQRHSGIKKKAQFPVFAQEECDKKWKNIEVIGEQLCAGGVFGIDSCSGDSGGPL**M**V
KRFYWIQEGVISFGNQCALEGWPGVYTRVSSYLDWIRQNIRR

Figure S4. ESI-MS analysis of covalent complexes formed by SRPN2 and CLIPB10$_{Xa}$ on SDS-PAGE. Signal peptides are italicized, sequence coverage is in red, peptide fragments are underlined, methionines with oxidation are bold. (**A**) Higher molecular band at ~72 kDa in Fig 2A. (**B**) Lower molecular band at ~55 kDa in Fig 2A.
