# Supplementary material for: CLIPB10 is a Terminal Protease in the Regulatory Network That Controls Melanization in the African Malaria Mosquito Anopheles gambiae
Source: Front Cell Infect Microbiol. 2021 Jan 15;10:585986. doi: 10.3389/fcimb.2020.585986 (PMC7843523; doi:10.3389/fcimb.2020.585986)
Supplement: Supplementary file 5 [file Table_1.docx]

# Table S1. Primer sequences

| Primer Function | Primer Name | Primer Sequences (5’-3’) | Description |
| --- | --- | --- | --- |
| ORF full-length cloning | CLIPB10_F | ATGCGGCCGCATGGCAAAGGTGGTGGATT | Added NotI site is underlined |
|  | CLIPB10_R | ACAAGCTTTCAATGGTGATGGTGATGATGACCTCCTCCACGACGAATGTTTTGCC | Added HindIII site is underlined. The reverse complement sequence for six histidines is double-underlined. |
| Mutagenesis | muCLIPB10_t | ACACGATCGAAGGACGGATCATCGGCGGTAACTACACGGCGA | Sequence encoding IEGR as replacement of wild-type LADR is underlined. |
| dsRNA synthesis | dsGFP_F | TAATACGACTCACTATAGGGCGATGC | Added sequence for T7 promoter is underlined |
|  | dsGFP_R | TAATACGACTCACTATAGGGCGGACT |  |
|  | dsSRPN2_F | CGACTCACTATAGGGCGAGGGCGCGGTCATTACG |  |
|  | dsSRPN2_R | CGACTCACTATAGGGCAGCATTGTTCCGAGGGTTTCATC |  |
|  | dsCLIPB10_F | TAATACGACTCACTATAGGGCCGCAAAACTTTGGCATC |  |
|  | dsCLIPB10_R | TAATACGACTCACTATAGGGCTGGTAGGCGGGAAGTCAT |  |
|  | dsCTL4_F | TAATACGACTCACTATAGGGGTTAGCAGCATTGGGATTACCCT |  |
|  | dsCTL4_R | TAATACGACTCACTATAGGGGAAGTCGCAACCCAGCTCATTGT |  |
|  | T7 | TAATACGACTCACTATAGGG | For second PCR |
| qPCR | qRPS7_F | GTGCGCGAGTTGGAGAAGA |  |
|  | qRPS7_R | ATCGGTTTGGGCAGAATGC |  |
|  | qSRPN2_F | TGCCGTGTCCAACACCAA |  |
|  | qSRPN2_R | CGCGTATGGTCGATGTTATCG |  |
|  | qCLIPB10_F | TTGATGGTAAAGCGGTTT |  |
|  | qCLIPB10_R | AGATACGACGACACTCTC |  |
