# Supplementary material for: CLIPB10 is a Terminal Protease in the Regulatory Network That Controls Melanization in the African Malaria Mosquito Anopheles gambiae
Source: Front Cell Infect Microbiol. 2021 Jan 15;10:585986. doi: 10.3389/fcimb.2020.585986 (PMC7843523; doi:10.3389/fcimb.2020.585986)
Supplement: Supplementary file 6 [file Table_2.docx]

**Table S2.** Malaria parasites infection in KD mosquitoes.

| **Exp. Group** | **Rep** | **Live Oocysts** | **Melanized parasites** | **Total parasites** | **Exp. Group** | **Rep** | **Live Oocysts** | **Melanized parasites** | **Total parasites** | **Exp. Group** | **Rep** | **Live Oocysts** | **Melanized parasites** | **Total parasites** | **Exp. Group** | **Rep** | **Live Oocysts** | **Melanized parasites** | **Total parasites** |
| --- | --- | --- | --- | --- | --- | --- | --- | --- | --- | --- | --- | --- | --- | --- | --- | --- | --- | --- | --- |
| LacZ | 1 | 0 | 0 | 0 | B10 | 1 | 1 | 0 | 1 | CTL4 | 1 | 0 | 2 | 2 | B10/CTL4 | 1 | 0 | 0 | 0 |
| LacZ | 1 | 13 | 0 | 13 | B10 | 1 | 2 | 0 | 2 | CTL4 | 1 | 0 | 32 | 32 | B10/CTL4 | 1 | 0 | 0 | 0 |
| LacZ | 1 | 16 | 0 | 16 | B10 | 1 | 8 | 0 | 8 | CTL4 | 1 | 2 | 53 | 55 | B10/CTL4 | 1 | 1 | 0 | 1 |
| LacZ | 1 | 17 | 0 | 17 | B10 | 1 | 11 | 0 | 11 | CTL4 | 1 | 12 | 73 | 85 | B10/CTL4 | 1 | 0 | 2 | 2 |
| LacZ | 1 | 38 | 0 | 38 | B10 | 1 | 14 | 0 | 14 | CTL4 | 1 | 1 | 100 | 101 | B10/CTL4 | 1 | 0 | 3 | 3 |
| LacZ | 1 | 45 | 0 | 45 | B10 | 1 | 17 | 0 | 17 | CTL4 | 1 | 4 | 102 | 106 | B10/CTL4 | 1 | 21 | 0 | 21 |
| LacZ | 1 | 47 | 0 | 47 | B10 | 1 | 19 | 0 | 19 | CTL4 | 1 | 16 | 103 | 119 | B10/CTL4 | 1 | 23 | 0 | 23 |
| LacZ | 1 | 48 | 0 | 48 | B10 | 1 | 24 | 0 | 24 | CTL4 | 1 | 3 | 141 | 144 | B10/CTL4 | 1 | 0 | 52 | 52 |
| LacZ | 1 | 49 | 0 | 49 | B10 | 1 | 28 | 0 | 28 | CTL4 | 1 | 0 | 150 | 150 | B10/CTL4 | 1 | 11 | 78 | 89 |
| LacZ | 1 | 51 | 0 | 51 | B10 | 1 | 30 | 0 | 30 | CTL4 | 1 | 0 | 193 | 193 | B10/CTL4 | 1 | 39 | 84 | 123 |
| LacZ | 1 | 53 | 4 | 57 | B10 | 1 | 32 | 0 | 32 | CTL4 | 1 | 0 | 255 | 255 | B10/CTL4 | 1 | 0 | 300 | 300 |
| LacZ | 1 | 73 | 0 | 73 | B10 | 1 | 35 | 0 | 35 | CTL4 | 1 | 7 | 353 | 360 | B10/CTL4 | 1 | 0 | 720 | 720 |
| LacZ | 1 | 89 | 0 | 89 | B10 | 1 | 45 | 0 | 45 | CTL4 | 1 | 2 | 454 | 456 | B10/CTL4 | 2 | 0 | 0 | 0 |
| LacZ | 1 | 89 | 0 | 89 | B10 | 1 | 66 | 0 | 66 | CTL4 | 1 | 5 | 625 | 630 | B10/CTL4 | 2 | 1 | 4 | 5 |
| LacZ | 1 | 108 | 0 | 108 | B10 | 1 | 72 | 0 | 72 | CTL4 | 1 | 0 | 660 | 660 | B10/CTL4 | 2 | 0 | 20 | 20 |
| LacZ | 1 | 114 | 0 | 114 | B10 | 1 | 76 | 0 | 76 | CTL4 | 1 | 7 | 868 | 875 | B10/CTL4 | 2 | 0 | 110 | 110 |
| LacZ | 1 | 129 | 0 | 129 | B10 | 1 | 109 | 0 | 109 | CTL4 | 2 | 1 | 36 | 37 | B10/CTL4 | 2 | 5 | 115 | 120 |
| LacZ | 1 | 180 | 0 | 180 | B10 | 1 | 119 | 0 | 119 | CTL4 | 2 | 0 | 151 | 151 | B10/CTL4 | 2 | 4 | 220 | 224 |
| LacZ | 1 | 188 | 0 | 188 | B10 | 1 | 120 | 0 | 120 | CTL4 | 2 | 6 | 192 | 198 | B10/CTL4 | 2 | 0 | 257 | 257 |
| LacZ | 2 | 0 | 0 | 0 | B10 | 1 | 163 | 0 | 163 | CTL4 | 2 | 16 | 292 | 308 | B10/CTL4 | 2 | 0 | 625 | 625 |
| LacZ | 2 | 0 | 0 | 0 | B10 | 1 | 191 | 0 | 191 | CTL4 | 2 | 2 | 335 | 337 | B10/CTL4 | 3 | 3 | 10 | 13 |
| LacZ | 2 | 0 | 0 | 0 | B10 | 1 | 62 | 339 | 401 | CTL4 | 2 | 0 | 350 | 350 | B10/CTL4 | 3 | 7 | 48 | 55 |
| LacZ | 2 | 10 | 0 | 10 | B10 | 2 | 1 | 0 | 1 | CTL4 | 2 | 6 | 527 | 533 | B10/CTL4 | 3 | 3 | 61 | 64 |
| LacZ | 2 | 12 | 4 | 16 | B10 | 2 | 4 | 0 | 4 | CTL4 | 2 | 0 | 698 | 698 | B10/CTL4 | 3 | 3 | 67 | 70 |
| LacZ | 2 | 15 | 3 | 18 | B10 | 2 | 5 | 0 | 5 | CTL4 | 3 | 2 | 5 | 7 | B10/CTL4 | 3 | 62 | 41 | 103 |
| LacZ | 2 | 30 | 0 | 30 | B10 | 2 | 7 | 0 | 7 | CTL4 | 3 | 6 | 2 | 8 | B10/CTL4 | 3 | 26 | 272 | 298 |
| LacZ | 2 | 36 | 2 | 38 | B10 | 2 | 23 | 0 | 23 | CTL4 | 3 | 1 | 8 | 9 | B10/CTL4 | 3 | 34 | 370 | 404 |
| LacZ | 2 | 66 | 0 | 66 | B10 | 2 | 56 | 0 | 56 | CTL4 | 3 | 11 | 0 | 11 | B10/CTL4 | 4 | 15 | 0 | 15 |
| LacZ | 2 | 76 | 0 | 76 | B10 | 2 | 67 | 0 | 67 | CTL4 | 3 | 24 | 18 | 42 | B10/CTL4 | 4 | 15 | 0 | 15 |
| LacZ | 2 | 95 | 0 | 95 | B10 | 2 | 148 | 0 | 148 | CTL4 | 3 | 3 | 117 | 120 | B10/CTL4 | 4 | 20 | 0 | 20 |
| LacZ | 2 | 114 | 0 | 114 | B10 | 2 | 191 | 0 | 191 | CTL4 | 3 | 0 | 125 | 125 | B10/CTL4 | 4 | 27 | 0 | 27 |
| LacZ | 2 | 117 | 0 | 117 | B10 | 2 | 234 | 0 | 234 | CTL4 | 3 | 12 | 415 | 427 | B10/CTL4 | 4 | 27 | 6 | 33 |
| LacZ | 2 | 137 | 0 | 137 | B10 | 2 | 450 | 0 | 450 | CTL4 | 3 | 4 | 563 | 567 | B10/CTL4 | 4 | 34 | 0 | 34 |
| LacZ | 2 | 171 | 0 | 171 | B10 | 3 | 2 | 0 | 2 | CTL4 | 3 | 7 | 806 | 813 | B10/CTL4 | 4 | 28 | 8 | 36 |
| LacZ | 2 | 165 | 11 | 176 | B10 | 3 | 14 | 0 | 14 | CTL4 | 3 | 3 | 824 | 827 | B10/CTL4 | 4 | 34 | 9 | 43 |
| LacZ | 2 | 183 | 1 | 184 | B10 | 3 | 17 | 0 | 17 | CTL4 | 4 | 84 | 0 | 84 | B10/CTL4 | 4 | 17 | 28 | 45 |
| LacZ | 2 | 276 | 0 | 276 | B10 | 3 | 19 | 0 | 19 | CTL4 | 4 | 33 | 82 | 115 | B10/CTL4 | 4 | 40 | 8 | 48 |
| LacZ | 2 | 383 | 0 | 383 | B10 | 3 | 19 | 0 | 19 | CTL4 | 4 | 72 | 63 | 135 | B10/CTL4 | 4 | 67 | 9 | 76 |
| LacZ | 3 | 0 | 0 | 0 | B10 | 3 | 17 | 11 | 28 | CTL4 | 4 | 100 | 59 | 159 | B10/CTL4 | 4 | 34 | 63 | 97 |
| LacZ | 3 | 0 | 0 | 0 | B10 | 3 | 35 | 0 | 35 | CTL4 | 4 | 11 | 280 | 291 | B10/CTL4 | 4 | 128 | 0 | 128 |
| LacZ | 3 | 1 | 0 | 1 | B10 | 3 | 35 | 0 | 35 | CTL4 | 4 | 1 | 370 | 371 | B10/CTL4 | 4 | 143 | 31 | 174 |
| LacZ | 3 | 14 | 0 | 14 | B10 | 3 | 51 | 0 | 51 | CTL4 | 4 | 20 | 380 | 400 | B10/CTL4 | 4 | 172 | 33 | 205 |
| LacZ | 3 | 15 | 0 | 15 | B10 | 3 | 53 | 0 | 53 | CTL4 | 4 | 7 | 408 | 415 | B10/CTL4 | 4 | 54 | 154 | 208 |
| LacZ | 3 | 53 | 0 | 53 | B10 | 3 | 89 | 0 | 89 | CTL4 | 4 | 5 | 537 | 542 | B10/CTL4 | 4 | 185 | 27 | 212 |
| LacZ | 3 | 136 | 0 | 136 | B10 | 3 | 95 | 0 | 95 | CTL4 | 4 | 7 | 599 | 606 | B10/CTL4 | 4 | 96 | 127 | 223 |
| LacZ | 3 | 167 | 0 | 167 | B10 | 3 | 105 | 0 | 105 | CTL4 | 4 | 2 | 613 | 615 | B10/CTL4 | 4 | 227 | 3 | 230 |
| LacZ | 3 | 200 | 0 | 200 | B10 | 3 | 111 | 0 | 111 | CTL4 | 4 | 4 | 851 | 855 | B10/CTL4 | 4 | 84 | 154 | 238 |
| LacZ | 3 | 225 | 0 | 225 | B10 | 3 | 150 | 0 | 150 | CTL4 | 4 | 11 | 1447 | 1458 | B10/CTL4 | 4 | 9 | 490 | 499 |
| LacZ | 4 | 0 | 0 | 0 | B10 | 3 | 166 | 0 | 166 | CTL4 | 5 | 1 | 3 | 4 | B10/CTL4 | 5 | 2 | 0 | 2 |
| LacZ | 4 | 0 | 0 | 0 | B10 | 3 | 170 | 0 | 170 | CTL4 | 5 | 39 | 0 | 39 | B10/CTL4 | 5 | 0 | 3 | 3 |
| LacZ | 4 | 45 | 0 | 45 | B10 | 3 | 200 | 0 | 200 | CTL4 | 5 | 0 | 40 | 40 | B10/CTL4 | 5 | 14 | 0 | 14 |
| LacZ | 4 | 56 | 0 | 56 | B10 | 3 | 222 | 0 | 222 | CTL4 | 5 | 35 | 14 | 49 | B10/CTL4 | 5 | 19 | 2 | 21 |
| LacZ | 4 | 64 | 0 | 64 | B10 | 4 | 0 | 0 | 0 | CTL4 | 5 | 24 | 30 | 54 | B10/CTL4 | 5 | 22 | 0 | 22 |
| LacZ | 4 | 72 | 0 | 72 | B10 | 4 | 30 | 0 | 30 | CTL4 | 5 | 48 | 14 | 62 | B10/CTL4 | 5 | 0 | 23 | 23 |
| LacZ | 4 | 75 | 0 | 75 | B10 | 4 | 21 | 17 | 38 | CTL4 | 5 | 4 | 113 | 117 | B10/CTL4 | 5 | 11 | 25 | 36 |
| LacZ | 4 | 166 | 0 | 166 | B10 | 4 | 44 | 0 | 44 | CTL4 | 5 | 1 | 181 | 182 | B10/CTL4 | 5 | 43 | 0 | 43 |
| LacZ | 4 | 178 | 0 | 178 | B10 | 4 | 45 | 0 | 45 | CTL4 | 5 | 3 | 243 | 246 | B10/CTL4 | 5 | 92 | 1 | 93 |
| LacZ | 4 | 208 | 0 | 208 | B10 | 4 | 60 | 0 | 60 | CTL4 | 5 | 0 | 281 | 281 | B10/CTL4 | 5 | 5 | 112 | 117 |
| LacZ | 4 | 295 | 0 | 295 | B10 | 4 | 61 | 0 | 61 | CTL4 | 5 | 0 | 500 | 500 | B10/CTL4 | 5 | 127 | 100 | 227 |
| LacZ | 5 | 38 | 0 | 38 | B10 | 4 | 75 | 0 | 75 | CTL4 | 5 | 0 | 510 | 510 | B10/CTL4 | 5 | 2 | 331 | 333 |
| LacZ | 5 | 41 | 0 | 41 | B10 | 4 | 80 | 0 | 80 | CTL4 | 5 | 0 | 648 | 648 |  |  |  |  |  |
| LacZ | 5 | 60 | 0 | 60 | B10 | 4 | 59 | 22 | 81 |  |  |  |  |  |  |  |  |  |  |
| LacZ | 5 | 61 | 0 | 61 | B10 | 4 | 82 | 0 | 82 |  |  |  |  |  |  |  |  |  |  |
| LacZ | 5 | 101 | 0 | 101 | B10 | 4 | 83 | 0 | 83 |  |  |  |  |  |  |  |  |  |  |
| LacZ | 5 | 107 | 0 | 107 | B10 | 4 | 88 | 0 | 88 |  |  |  |  |  |  |  |  |  |  |
| LacZ | 5 | 133 | 0 | 133 | B10 | 4 | 107 | 0 | 107 |  |  |  |  |  |  |  |  |  |  |
| LacZ | 5 | 131 | 28 | 159 | B10 | 4 | 125 | 0 | 125 |  |  |  |  |  |  |  |  |  |  |
| LacZ | 5 | 162 | 0 | 162 | B10 | 4 | 134 | 0 | 134 |  |  |  |  |  |  |  |  |  |  |
| LacZ | 5 | 181 | 0 | 181 | B10 | 4 | 136 | 0 | 136 |  |  |  |  |  |  |  |  |  |  |
|  |  |  |  |  | B10 | 4 | 231 | 0 | 231 |  |  |  |  |  |  |  |  |  |  |
|  |  |  |  |  | B10 | 4 | 244 | 0 | 244 |  |  |  |  |  |  |  |  |  |  |
|  |  |  |  |  | B10 | 4 | 311 | 0 | 311 |  |  |  |  |  |  |  |  |  |  |
|  |  |  |  |  | B10 | 4 | 343 | 0 | 343 |  |  |  |  |  |  |  |  |  |  |
|  |  |  |  |  | B10 | 5 | 0 | 0 | 0 |  |  |  |  |  |  |  |  |  |  |
|  |  |  |  |  | B10 | 5 | 0 | 0 | 0 |  |  |  |  |  |  |  |  |  |  |
|  |  |  |  |  | B10 | 5 | 1 | 0 | 1 |  |  |  |  |  |  |  |  |  |  |
|  |  |  |  |  | B10 | 5 | 2 | 0 | 2 |  |  |  |  |  |  |  |  |  |  |
|  |  |  |  |  | B10 | 5 | 3 | 0 | 3 |  |  |  |  |  |  |  |  |  |  |
|  |  |  |  |  | B10 | 5 | 3 | 0 | 3 |  |  |  |  |  |  |  |  |  |  |
|  |  |  |  |  | B10 | 5 | 2 | 1 | 3 |  |  |  |  |  |  |  |  |  |  |
|  |  |  |  |  | B10 | 5 | 12 | 1 | 13 |  |  |  |  |  |  |  |  |  |  |
|  |  |  |  |  | B10 | 5 | 26 | 2 | 28 |  |  |  |  |  |  |  |  |  |  |
|  |  |  |  |  | B10 | 5 | 39 | 4 | 43 |  |  |  |  |  |  |  |  |  |  |
|  |  |  |  |  | B10 | 5 | 44 | 0 | 44 |  |  |  |  |  |  |  |  |  |  |
|  |  |  |  |  | B10 | 5 | 54 | 2 | 56 |  |  |  |  |  |  |  |  |  |  |
|  |  |  |  |  | B10 | 5 | 66 | 0 | 66 |  |  |  |  |  |  |  |  |  |  |
|  |  |  |  |  | B10 | 5 | 90 | 0 | 90 |  |  |  |  |  |  |  |  |  |  |
|  |  |  |  |  | B10 | 5 | 104 | 0 | 104 |  |  |  |  |  |  |  |  |  |  |
