# Supplementary material for: CLIPB10 is a Terminal Protease in the Regulatory Network That Controls Melanization in the African Malaria Mosquito Anopheles gambiae
Source: Front Cell Infect Microbiol. 2021 Jan 15;10:585986. doi: 10.3389/fcimb.2020.585986 (PMC7843523; doi:10.3389/fcimb.2020.585986)
Supplement: Supplementary file 7 [file Table_3.docx]

**Table S3.** Summary statistics of live oocyst and melanized ookinete load, respectively as shown in Figure 1C. Data were analyzed using the Kruskal-Wallis test, followed by Dunn's multiple comparisons test.

| Test details | Post test *P* | Mean rank 1 | Mean rank 2 | Mean rank diff. | n1 | n2 |
| --- | --- | --- | --- | --- | --- | --- |
| **Live oocysts *P* < 0.0001** |  |  |  |  |  |  |
| *LacZ* vs. *B10* | > 0.05 | 160.4 | 134.2 | 26.20 | 61 | 85 |
| *LacZ* vs. *CTL4* | < 0.001 | 160.4 | 56.03 | 104.4 | 61 | 46 |
| *LacZ* vs*. CTL4*/*B10* | < 0.001 | 160.4 | 101.5 | 58.98 | 61 | 46 |
| *B10* vs. *CTL4* | < 0.001 | 134.2 | 56.03 | 78.20 | 85 | 46 |
| *B10* vs. *CTL4*/*B10* | > 0.05 | 134.2 | 101.5 | 32.78 | 85 | 46 |
| *CTL4* vs. *CTL4*/*B10* | < 0.01 | 56.03 | 101.5 | -45.42 | 46 | 46 |
| **Melanized Okinetes P < 0.0001** | |  |  |  |  |  |
| *LacZ vs. B10* | > 0.05 | 18.79 | 25.44 | -6.659 | 7 | 9 |
| *LacZ vs. CTL4* | < 0.001 | 18.79 | 75.90 | -57.11 | 7 | 58 |
| *LacZ vs. CTL4/B10* | > 0.05 | 18.79 | 51.33 | -32.54 | 7 | 44 |
| *B10 vs. CTL4* | < 0.001 | 25.44 | 75.90 | -50.45 | 9 | 58 |
| *B10 vs. CTL4/B10* | > 0.05 | 25.44 | 51.33 | -25.89 | 9 | 44 |
| *CTL4 vs. CTL4/B10* | < 0.001 | 75.90 | 51.33 | 24.57 | 58 | 44 |
