# Supplementary material for: CLIPB10 is a Terminal Protease in the Regulatory Network That Controls Melanization in the African Malaria Mosquito Anopheles gambiae
Source: Front Cell Infect Microbiol. 2021 Jan 15;10:585986. doi: 10.3389/fcimb.2020.585986 (PMC7843523; doi:10.3389/fcimb.2020.585986)
Supplement: Supplementary file 8 [file Table_4.docx]

**Table S4.** Scores of parasite infection and statistical evaluation of prevalence. Number of guts with live oocysts was the sum of guts with live oocysts only and guts with both live and melanized parasites. Number of guts with melanized ookinetes was the sum of guts with melanized ookinetes only and guts with both live and melanized parasites.

|  | LacZ | CLIPB10 | CLT4 | CTL4/B10 |
| --- | --- | --- | --- | --- |
| Total # of guts | 69 | 88 | 61 | 60 |
| # with live oocysts only | 54 | 76 | 3 | 13 |
| # with melanized ookinetes only | 0 | 0 | 15 | 11 |
| # with both live and melanized parasites | 7 | 9 | 43 | 33 |
| # without any parasites | 8 | 3 | 0 | 3 |
| # with live oocysts | 61 | 85 | 46 | 46 |
| # of the rest | 8 | 3 | 15 | 14 |
|  | *p* = 0.0257* | *p* < 0.0001* | *p* = 0.2905* |  |
| # with melanized ookinetes | 7 | 9 | 58 | 44 |
| # of the rest | 62 | 79 | 3 | 16 |
|  | *p* < 0.0001* | *p* < 0.0001* | *p* =0.0003* |  |

* *χ*^2^ test; each treatment group was compared to the *CTL4/B10* treatment, followed by Bonferroni correction.
